# Mining the key genes for ventilator-induced lung injury using co-expression network analysis

**DOI:** 10.1042/BSR20203235

**Published:** 2021-03-17

**Authors:** Zhao Li, Yajun Xiao, Li Xu, Qingxiu Wang

**Affiliations:** 1 Department of Anesthesiology, East Hospital, Tongji University School of Medicine, Shanghai, P.R. China; 2 Department of Endocrinology, Affiliated Nanhua Hospital, University of South China, Hengyang, P.R. China; 3 Department of Anesthesiology, The First People’s Hospital of Changde, Changde, P.R. China

**Keywords:** Bioinformatics, VILI, WGCNA

## Abstract

Mechanical ventilation is extensively adopted in general anesthesia and respiratory failure management, but it can also induce ventilator-induced lung injury (VILI). Therefore, it is of great urgency to explore the mechanisms involved in the VILI pathogenesis, which might contribute to its future prevention and treatment. Four microarray datasets from the GEO database were selected in our investigation, and were subjected to the Weighted Gene Co-Expression Network Analysis (WGCNA) to identify the VILI-correlated gene modules. The limma package in R software was used to identify the differentially expressed genes (DEGs) between the VILI and control groups. WGCNA was constructed by merging the GSE9314, GSE9368, GSE11434 and GSE11662 datasets. A total of 49 co-expression network modules were determined as associated with VILI. The intersected genes between hub genes screened from DEGs for VILI and those identified using WGCNA were as follows: Tlr2, Hmox1, Serpine1, Mmp9, Il6, Il1b, Ptgs2, Fos and Atf3, which were determined to be key genes for VILI. Those key genes were validated by GSE86229 and quantitative PCR (qPCR) experiment to have significantly statistical difference in their expression between the VILI and control groups. In a nutshell, nine key genes with expression differences in VILI were screened by WGCNA by integrating multiple datasets.

## Background

Mechanical ventilation is a therapeutic measure usually taken in dealing with general anesthesia and respiratory failure [1,2]. However, it has been increasingly manifested that using mechanical ventilation in respiratory care has some tradeoffs [3]. Other than providing respiratory support, it could also induce or aggravate lung injuries, known as Ventilator-Induced Lung Injury (VILI) [4]. Gajic et al. found that within 3 days after being given at least 48-h mechanical ventilation, 25% of patients originally diagnosed as having no acute lung injury (ALI) developed symptoms of ALI [5]; when mechanical ventilation lasted for more than 2 weeks, nearly half of the patients showed pulmonary complications associated with mechanical ventilation [6]. Therefore, there is a pressing need to find means to effectively prevent and treat VILI.

A further study on the mechanisms of VILI revealed that the main cause of VILI is mechanical injury [4]. The current practice in the VILI prevention is the adoption of low tidal volume ventilation, whose efficacy is limited and dubious [7]. Apart from being caused by mechanical impairment, there is another type of lung injury induced by the massive release of inflammatory factors in the lung, known as biological injury [8,9]. It is currently uncertain about the pathological mechanism of such injury, and its effective intervening measures are wanting [10]. And if key genes involved in such injury are identified, it will greatly contribute to better understanding of molecular mechanism of VILI and help to take an early interference in such injury.

The molecular and genetic basis of VILI has been investigated comprehensively by a large number of studies, and it has been found that gene activation plays an important role in the pathophysiological process of VILI [11]. Bioinformatics analysis, a new approach to studying gene function, has also been used to analyze the molecular mechanisms of VILI, but mainly in terms of differentially expressed genes (DEGs). Dolinay et al. analyzed the gene expression profiles in a VILI mouse model and validated five up-regulated genes [12]. Ma et al. investigated VILI-associated genes and validated the expression of 15 randomly selected genes [13]. In another study, Xia et al. revealed that non-canonical WNT signaling could participate in the VILI development [14]. Although the significance of such studies is obvious, limitations imposed by factors such as sequencing costs lead to inclusion of small sample sizes in a single dataset in those studies. Therefore, integrating the data already present in the GEO database and expanding the sample size for analysis are necessary to make the best use of these resources. To our best knowledge, there has been no studies on the VILI progression using the weighted gene co-expression network analysis (WGCNA) and hub genes associated with VILI.

In the present study, we derived microarray data of gene expression profiles of mouse lung tissues from the GEO database, and integrated four expression profiling datasets to construct expression network for WGCNA, to explore the dynamic process of VILI pathogenesis and development. The patterns of expression profiles in the VILI and control groups and the differences between these profiles were comprehensively analyzed by using WGCNA and other specialized bioinformatics analysis tools. The results concluded in the present study could be conducive to comprehensively understanding the VILI pathogenesis, pinpointing the molecular mechanism involved in the pathological process and providing insights into novel treatment and therapeutic targets for drugs.

## Materials and methods

### Gene expression data acquisition

Gene Expression Omnibus (GEO) is currently the most comprehensive public gene expression database that incorporates high-throughput sequencing, gene expression microarray, and other genomics data. In the present study, series matrix files of datasets, including GSE9314, GSE9368, GSE11434 and GSE11662, were exported from the GEO database to construct WGCNA. These four datasets were all based on the GPL1261 platform (Array type: [Mouse430_2] Affymetrix Mouse Genome 430 2.0 Array; Experiment type: expression profiling by array; organism: *Mus musculus*). GSE9314 included eight transcriptome datasets for the VILI (*n*=4) and control (*n*=4) groups; GSE9368 included six transcriptome datasets for the VILI (*n*=3) and control (*n*=3) groups, GSE11434 included ten transcriptome datasets for the VILI (*n*=5) and control (*n*=5) groups, while GSE11662 comprised six transcriptome datasets for the VILI (*n*=3) and control (*n*=3) groups. GSE86229 was used to validate the result of WGCNA. GSE86229 was based on GPL6246 platform (Array type: [MoGene-1_0-st] Affymetrix Mouse Genome 1.0 ST Array; Experiment type: expression profiling by array; organism: *Mus musculus*). The downloaded microarrays were converted into gene symbols through annotations in the platform annotation files.

### WGCNA

The WGCNA package for R (ver. 3.6.2) was used to construct weighted gene co-expression network, identify the co-expressed gene modules, explore the correlation between the gene network and biological traits, and investigate hub genes in the network. At first, the combined matrix files were transposed to screen out genes whose expression variance was within the first quartile, and the correlation matrix was constructed subsequently. The Pearson correlation coefficient between genes was then calculated, and the filtering threshold was applied to determine whether the genes had similar expression profiles. Then the weighted adjacency matrix was converted into topological overlap matrix (TOM) to evaluate the network connectivity, and the hierarchical clustering method was used to construct clustering dendrograms of TOM. Nodes of the dendrogram and colors stand for different corresponding gene modules. Based on the weighted correlation coefficient, genes were clustered into different modules according to their expression patterns, i.e. genes with similar expression patterns were grouped into the same module. After genes were clustered, heatmap was plotted to visualize and calculate the intermodule correlation. The correlation between modules and clinical traits was further evaluated to determine the modules associated with VILI for analysis.

### Functional enrichment analysis of gene modules

The intramodular genes most significantly correlated with VILI from WGCNA results were loaded into the Metascape database for enrichment analysis. Metascape is an effective tool designed to provide comprehensive gene annotations and analysis resources [15]. GO enrichment analysis and pathway enrichment analysis of the acquired genes were performed with the screening condition set at *P*<0.01 for statistical significance, the min overlap set at 3 and the min enrichment set at 1.5.

### Identification of DEGs

Differential expression analysis was performed based on the merged expression profiling data, and DEGs were identified using the limma package for R, under the condition of adj *P*<0.05 and |log_2_FC|>1. The identified DEGs were used for volcano plotting.

### Protein–protein interaction analysis and hub gene identification

Genes at the center of the regulatory network are defined as hub genes. To identify the hub genes in the VILI-correlated modules, we constructed a protein–protein interaction (PPI) network, which is a gene-relationship graph based on the information of each intramodular gene. Then Cytoscape was used to visualize the network, and the cytohubba plug-in was used to screen out hub genes. The value of each gene was computed using the five topological algorithms (Betweenness, Closeness, Degree, MNC and Stress), and the top 20 genes were intersected to identify the hub genes in DEGs. The MCODE plug-in was used to detect significant subnetworks in the PPI network.

### Animal grouping and VILI model construction

A total of ten healthy male ICR mices were purchased from Shanghai JSJ-Lab, and randomized into two groups: the VILI group and control group. Animal care and all experimental procedures were conducted in the Tongji University, School of Medicine (Shanghai, China) and according to the guidelines for the care and use of experimental animals of Tongji University (Shanghai, China). The animal protocols were approved by the Animal Ethics Committee of Tongji University, School of Medicine (Shanghai, China). Mices were abstained from food 4 h before VILI model construction, and were anesthetized via intraperitoneal injection of 75 mg/kg sodium pentobarbital (MERCK, Germany). Then mice were anesthetized and subjected to tracheal intubation. The mechanical ventilation parameters were set at: tidal volume of 30 ml/kg, 65 breaths/min and fraction of inspired oxygen of 0.21. The ventilation process lasted for 4 h. Nonmechanically ventilated mice were used as controls.

### RNA extraction and quantitative PCR

At the end of the experiment, all animals were killed via sodium pentobarbital at a dose of 150 mg/kg and lung tissues were isolated. TRIzol reagent (Takara, Japan) was used to extract the total RNA from mice lung tissues. cDNA was reverse-transcribed from 1 μg equivalent RNA using Reverse Transcription Kit (Takara, Japan). The SYBR Green method (Qiagen, Germany) was used to perform quantitative PCR (qPCR). Then the cDNA was used as templates to perform PCR for 40 cycles under the following conditions: at 95°C for 20 s of initial denaturation, at 95°C for 1 s of denaturation and at 60°C for 20 s of annealing. The following primers for cDNA sequences were used in PCR: GAPDH forward, 5′-AGGTCGGTGTGAACGGATTTG-3′ and reverse, 5′-TGTAGACCATGTAGTTGAGGTCA-3′; Activating Transcription Factor 3 (ATF3) forward, 5′-GAGGATTTTGCTAACCTGACACC-3′ and reverse, 5′-TTGACGGTAACTGACTCCAGC-3′; FOS forward, 5′-CGGGTTTCAACGCCGACTA-3′ and reverse, 5′-TTGGCACTAGAGACGGACAGA-3′; Heme oxygenase 1 (HMOX1) forward, 5′- AAGCCGAGAATGCTGAGTTCA-3′ and reverse, 5′- GCCGTGTAGATATGGTACAAGGA-3′; IL-6 forward, 5′-TAGTCCTTCCTACCCCAATTTCC-3′ and reverse, 5′-TTGGTCCTTAGCCACTCCTTC-3′; IL-1B forward, 5′-GCAACTGTTCCTGAACTCAACT-3′ and reverse, 5′-ATCTTTTGGGGTCCGTCAACT-3′; matrix metalloproteinase (MMP) 9 (MMP9) forward, 5′-CTGGACAGCCAGACACTAAAG-3′ and reverse, 5′-CTCGCGGCAAGTCTTCAGAG-3′; prostaglandin-endoperoxide synthase 2 (PTGS2) forward, 5′-TTCAACACACTCTATCACTGGC-3′ and reverse, 5′-AGAAGCGTTTGCGGTACTCAT-3′; SERPINE1 forward, 5′-TTCAGCCCTTGCTTGCCTC-3′ and reverse, 5′-ACACTTTTACTCCGAAGTCGGT-3′; toll-like receptor (TLR) 2 (TLR2) forward, 5′-ATGGCATGGCTTACACCACC-3′ and reverse, 5′-GAGGCCAATTTTGTCTCCACA-3′. The amplification procedures were conducted with a QuantStudio 7 flex real-time PCR system. The normalized mRNA expressions were expressed as ratios of Tlr2, Hmox1, Serpine1, Mmp9, Il6, Il1b, Ptgs2, Fos and Atf3 respectively against the reference gene Gapdh. The cycle threshold (*C*_T_) values were read through quantitative fluorescence PCR to determine the fold change of expression of each target gene against the control.

### Statistical analysis

R (version 3.6.2) was utilized for statistical analysis and plotting in the present study. All statistical tests were two-sided and met the condition of *P*<0.05.

## Results

### Data preprocessing before analysis

GSE datasets were downloaded, and probe IDs were converted into gene symbols in the PERL. Probes matching multiple genes were removed from the datasets. And in the cases of multiple probes for one gene, the average expression value of probes was taken as the gene expression level. The expression microarray data from the four datasets were merged in the PERL, and matrix files containing 20814 genes were obtained subsequently. When merging the data, we only preserved the expressions of genes that were detected in all microarrays. If a gene was detected only in the microarray platform where a particular dataset was located, but not present in others, then its data were excluded, and no further analysis would be performed on it. ComBat function in the sva package was utilized in R to adjust for batch effect. Final expression matrix was obtained after batch correction, which was composed of the mRNA expression data, and was directly used in subsequent analyses.

### Power law (β) selection and scale-free network construction

**Figure 1:**
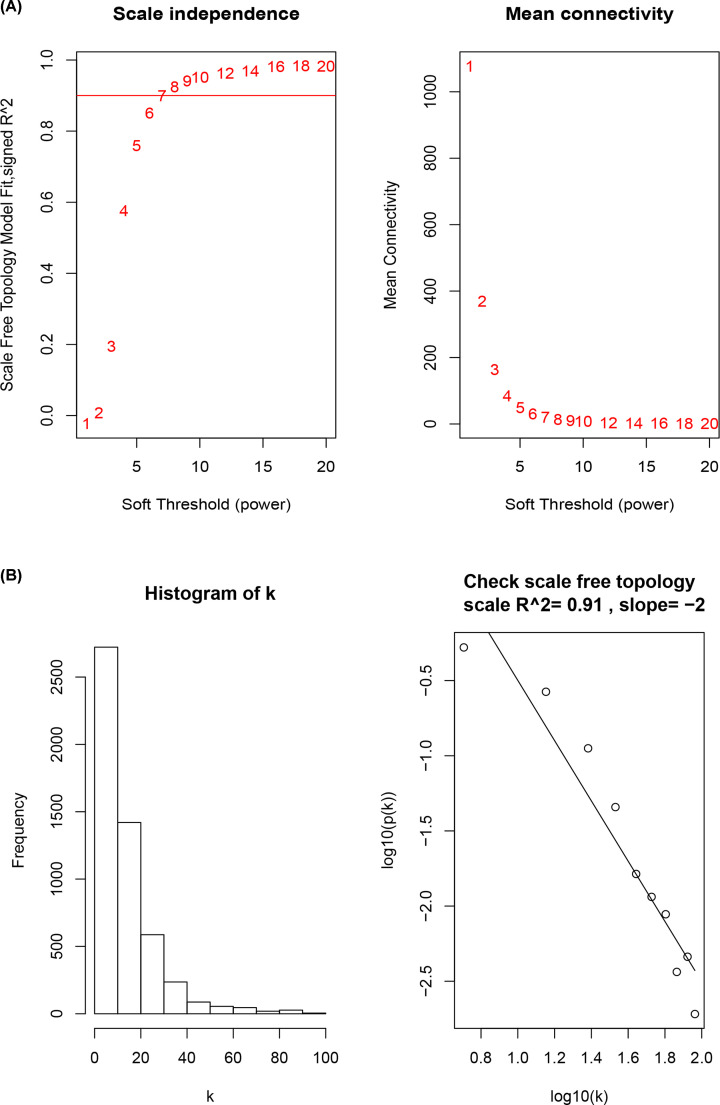
Scale-free network construction in the co-expression network (**A**) Scale independence and mean connectivity analysis. (**B**) Analysis of connectivity distribution and scale-free topology when β = 8.

No outliers were detected in the expression matrix consisting of the expressions of 5203 genes, whose expression variance fell within the first quartile, in samples of the control and VILI groups. Pearson correlation coefficient was used to calculate the correlation coefficient of expression between genes to construct the correlation matrix. And then the power value β was calculated and selected; that is, the probability p(k) of nodes with connectivity k was fitted to lg(p(k)), and when the fitting index reached 0.9, the β value, which maximized the average connectivity (the mean value of k), was selected to perform a power-law operation to convert the correlation matrix into adjacency matrix. The β values were set between 1 and 20, respectively, to fit with the scale-free network, and to calculate the corresponding fitting index and average connectivity for different β values, as shown in Figure 1A. When the β value was at 8, the highest average connectivity in the network could be achieved with a fitting index > 0.9. As shown in Figure 1B, the network met the patten of a scale-free network when the β value reached 8. Therefore, in the present study, the β value of 8 was adopted in constructing the scale-free network, which was successfully constructed after converting the correlation matrix into adjacency matrix.

### Construction of TOM and identification of co-expression modules

**Figure 2:**
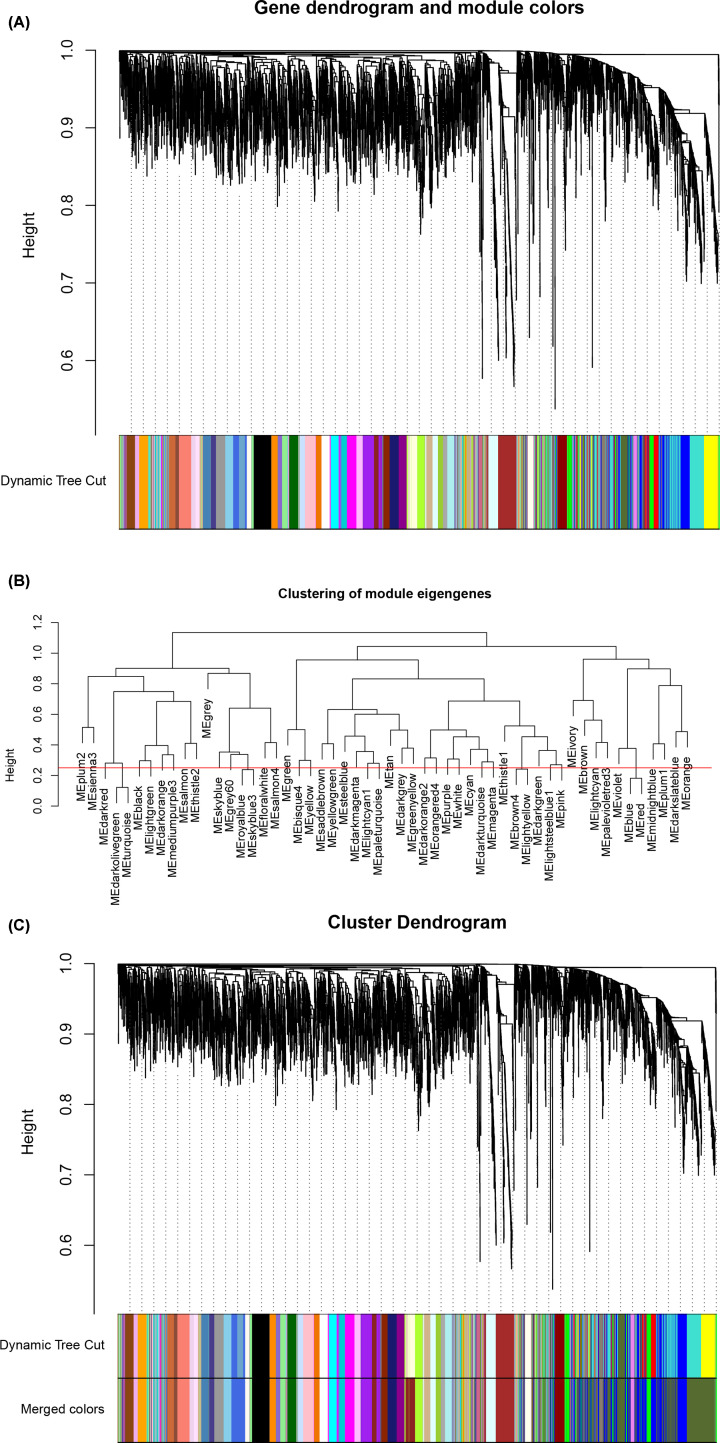
Module identification with WGCNA (**A**) Primary results of clustering tree construction and module identification. (**B**) Merging of modules with similar expression profiles. (**C**) Network dendrogram based on differential measurement and identified module colors. In the dendrogram of (A) and (C), each node represents a gene, and the vertical axis denotes the degree of topological differences between genes, i.e. a larger distance between vertical coordinates indicates a larger topological difference, meaning a weaker co-expression correlation; the horizontal axes represent different modules, with each color denoting a module and the width of color bar indicating the gene number in the module; Dynamic Tree Cut represents modules initially obtained through average-linkage hierarchical clustering. Merged colors denote the reconstructed modules after merging similar modules.

The adjacency matrix was converted into TOM based on the topological overlap, and the clustering method was used to cluster genes with high topological overlap to construct a clustering dendrogram, which was then pruned using the dynamic tree cut method to further classify the modules, so that genes with high topological overlap were clustered into the same module. The number of initially classified modules was relatively larger (Figure 2A), indicating that some genes with high similarity were not clustered together. Considering excessive module number would be difficult for subsequent analysis, the module eigengenes in each module were subjected to clustering analysis, and the similarity threshold was set to 0.25 to cluster similar modules into one (Figure 2B).

Based on the co-expression correlation, the total 5203 genes were clustered into 49 gene co-expression modules, which had different numbers of genes, with the largest one containing 701 genes and the smallest containing 33 genes (Figure 2C). It was shown from the result that the darkolivegreen module was the largest, including 701 genes, which were represented as a large branch in the dendrogram; this module had small topological variations between the intramodular genes (closer in distance between coordinates), but had much larger topological variations from the genes of other modules on different branches; moreover, merged colors indicated that the darkolivegreen module had great gene consistency, with few genes of other module mixed in it. Other modules were all of similar module size, each branch had a clear outline, and modules with the same color showed excellent gene consistency, all suggesting that construction of scale-free networks, construction of TOM and module classification results were all reliable. The following section will further validate the effectiveness and reliability of the module construction.

### Validation of module construction, module–trait association analysis and trait-associated modules identification

**Figure 3:**
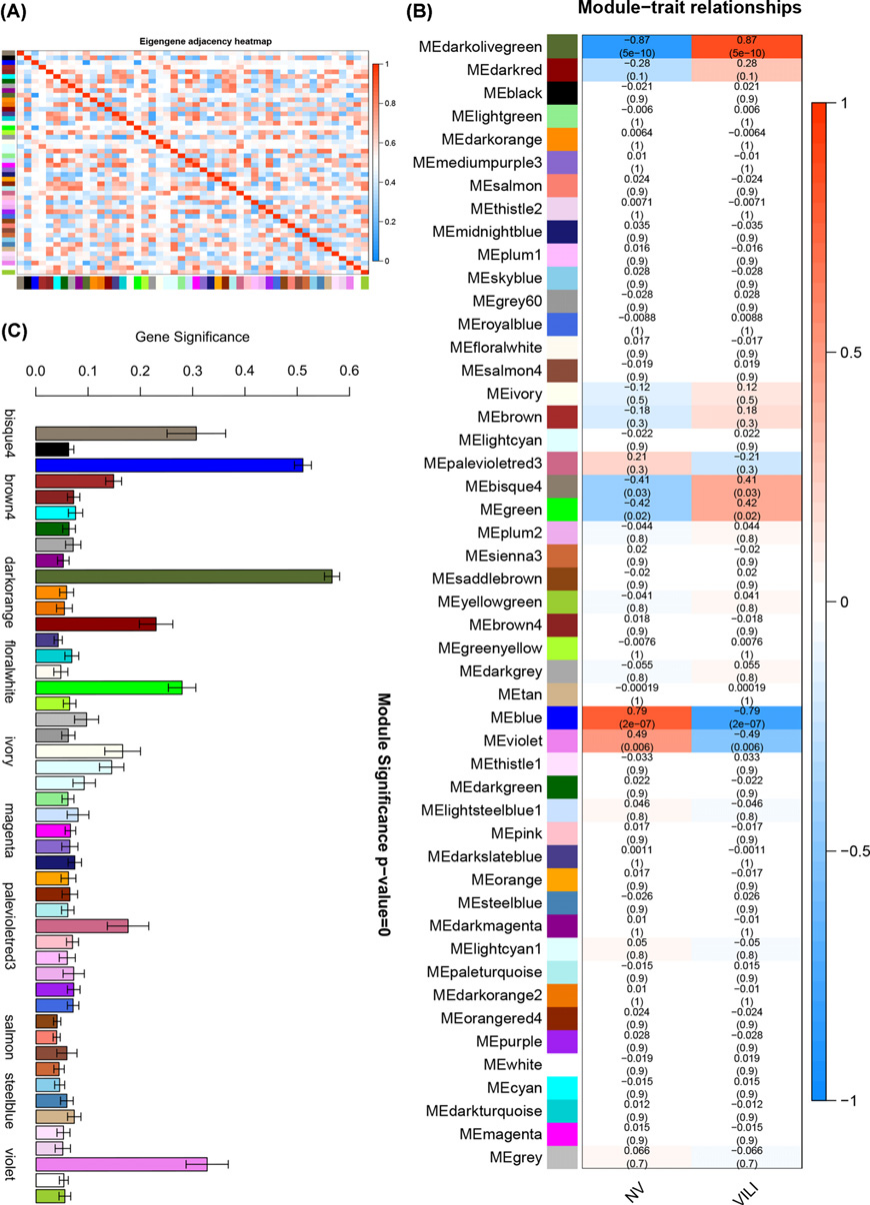
Correlation of module eigengenes and module–trait correlation (**A**) Heatmap for module eigengenes. The correlation of module eigengenes increases gradually with color changing from blue to red. (**B**) Heatmap for the correlation between modules and VILI traits. The horizontal axes represent different modules. The color of each cell indicates the corresponding module–trait correlation, a deeper red color suggesting stronger positive correlation and a deeper blue color suggesting stronger negative correlation. The value in each cell denotes the correlation score, and the value in the bracket below denotes the significance (*P*-value). (**C**) Module significance associated with VILI.

The modules were constructed based on the topological overlap, and intramodular genes should have high correlation with genes within the module and low correlation with other extramodular genes; however, given that a total of 5203 genes were classified, and soft-threshold power was selected to distinguish between modules instead of hard threshold, it was likely that the intramodular genes were correlated with extramodular genes. The genes with the highest gene significance (GS) and module membership (MM) in the module were defined as module eigengenes, and heatmap of module eigengenes was plotted according to the significance of correlations between them defined by Pearson correlation coefficient, as shown in Figure 3A. It can be seen in Figure 3A that the red blocks were mainly concentrated on the upper left and lower right diagonals, and the other regions were dominated by blue blocks, indicating that the module eigengenes in different modules were not highly correlated with each other (i.e., the module eigengenes could serve as representative of the profiles of the corresponding module genes) and there was no significant co-expression correlation with the module eigengenes of other modules, reflecting strong independence between different modules. It was revealed that the module classification was accurate and reliable, and could be used in further single module analysis.

Based on the constructed modules, the correlation between the module and traits was analyzed and its significance was calculated; the calculated results were used to plot the module–trait relationship heatmap (Figure 3B). As shown in Figure 3B, the darkolivegreen and blue modules exhibited significant association with VILI traits, indicating their role in the pathogenesis and progression of VILI. As shown in Figure 3C, the absolute values of GS in the darkolivegreen and blue modules were the highest among all modules, further confirming the significant correlation between the two modules and VILI.

### Gene analysis in trait-associated modules

**Figure 4:**
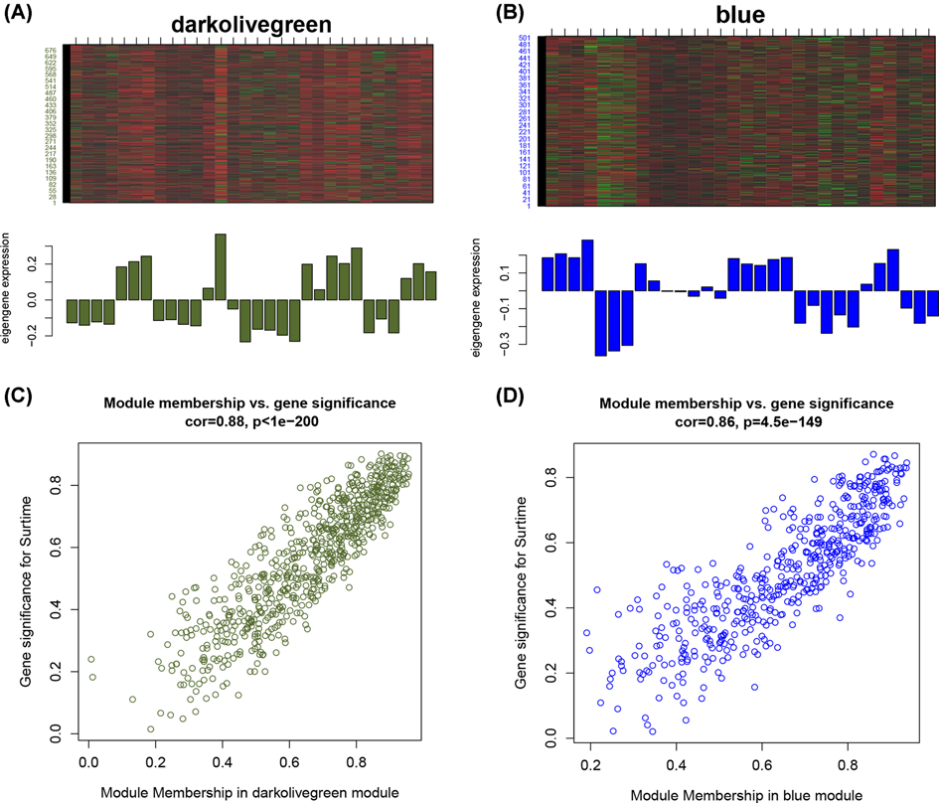
The analysis of the module most related to VILI The heatmap and histogram of gene expression patterns in the darkolivegreen (**A**) and blue (**B**) modules. The scatter plot of module eigengenes in the darkolivegreen (**C**) and blue (**D**) modules.

Heatmap for the darkolivegreen and blue modules is shown in Figure 4A–D. The darkolivegreen module contained 701 genes, and had a correlation coefficient of 0.87 with VILI traits, with a significance of less than 0.01; the blue module included 505 genes, and had a correlation coefficient of −0.79 with VILI traits, with a significance of less than 0.01. Based on the condition of GS > 0.2 and MM > 0.8, 223 hub genes and 219 hub genes were identified in the darkolivegreen module and blue module, respectively.

### Functional enrichment analysis of VILI-correlated modules

**Figure 5:**
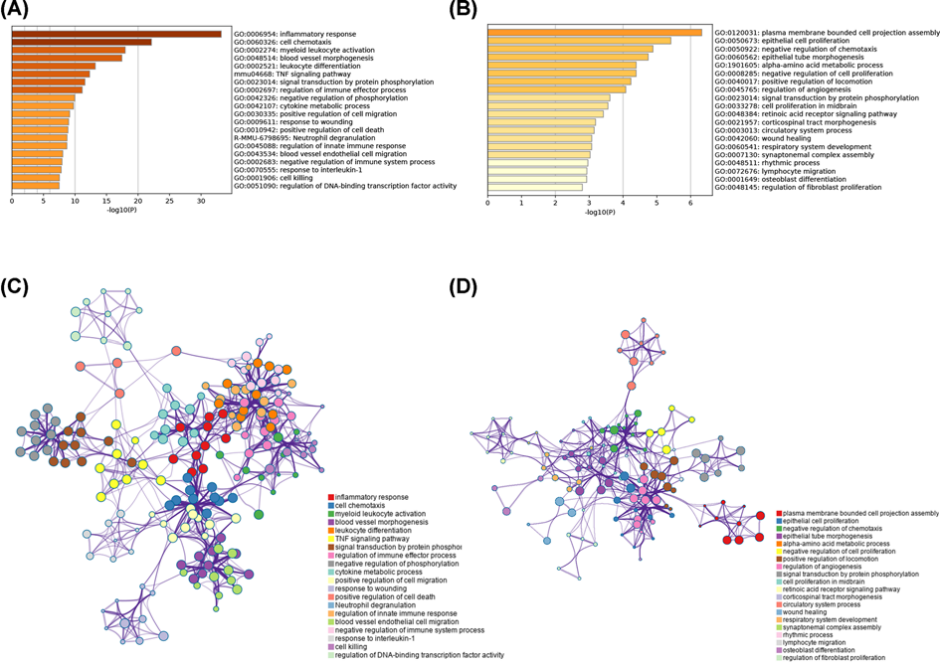
Enrichment analysis of the module most related to VILI Heatmap of enriched terms in the darkolivegreen (**A**) and blue (**B**) modules. Network of enriched terms colored by *P*-value in the darkolivegreen (**C**) and blue (**D**) modules.

In order to determine the biological functions and signaling pathways for the two modules, the Metascape database was used for annotation and visualization. The result showed that the darkolivegreen module was primarily enriched for the inflammatory response and regulation of innate immune response (Figure 5A,B). The blue module was also enriched for immune-related functions, suggesting that the immune response plays an important role in VILI pathology (Figure 5C,D).

### DEGs identification

**Figure 6:**
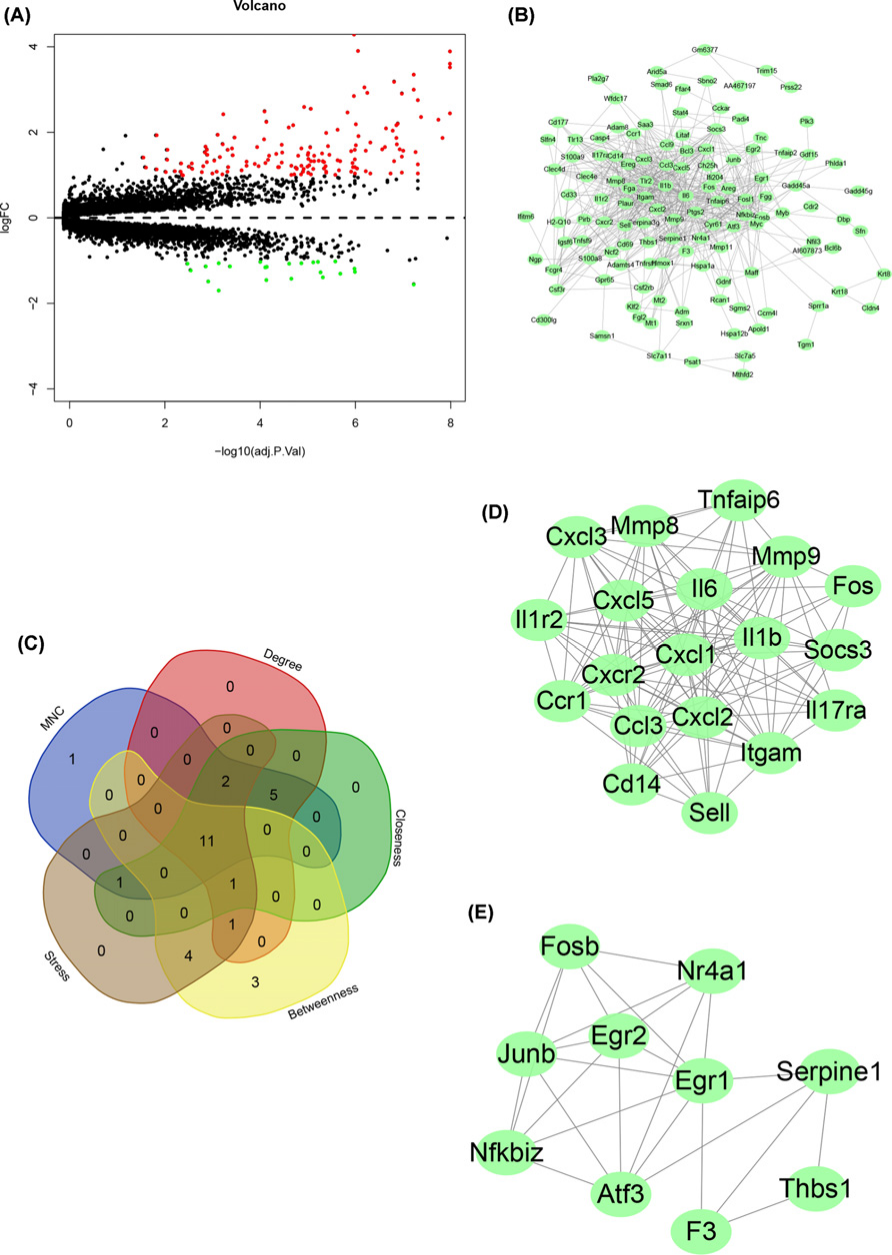
The analysis of DEGs in VILI (**A**) Volcano plot representing VILI-related DEGs. (**B**) PPI network of VILI-related DEGs. (**C**) Intersecting genes selected as hub genes by using five algorithms in CytoHubba. (**D**) The most significant module among PPI network. (**E**) The second most significant module among PPI network.

By applying the eBayes function in the limma package, differential expression analysis was performed based on the Bayes approach in R. The logFC was set at |log_2_ FC| > 1, and the significance threshold was set at adj *P*<0.05. A total of 148 genes were up-regulated and 23 were down-regulated; the differential expression volcano plot of 171 genes was constructed (Figure 6A).

### PPI

The corresponding proteins for the 171 DEGs between the control and VILI groups were selected for PPI analysis, using the STRING database and its relevant analysis tools. The interaction score threshold was set at 0.4 for this analysis, i.e. an overall score of less than 0.4 was considered as indicative of no interaction. Proteins meeting this scoring criterion were selected and their interactions were visualized by Cytoscape. Proteins independent from the interaction network were removed, and those, forming more and stronger interactions pairs with others, were preserved for plotting the PPI network graph, as shown in Figure 6B. The information such as the position of a certain protein in the network, its interaction intensity with other proteins and number of interactions with other proteins can be obtained from Figure 6B. Five algorithms, Betweenness, Closeness, Degree, MNC and Stress for CytoHubba were used to identify hub genes in the PPI network, and top 20 genes sorted by each algorithm were selected for interaction testing, which yielded 11 hub genes: Serpine1, Fos, Itgam, Mmp9, Il6, Hmox1, Tlr2, Egr1, Atf3, Ptgs2 and Il1b (Figure 6C). Based on the interaction pair number and interaction intensity, further clustering was performed using MCODE to construct a subnetwork, as shown in Figure 6D,E.

### Key genes for VILI

**Figure 7:**
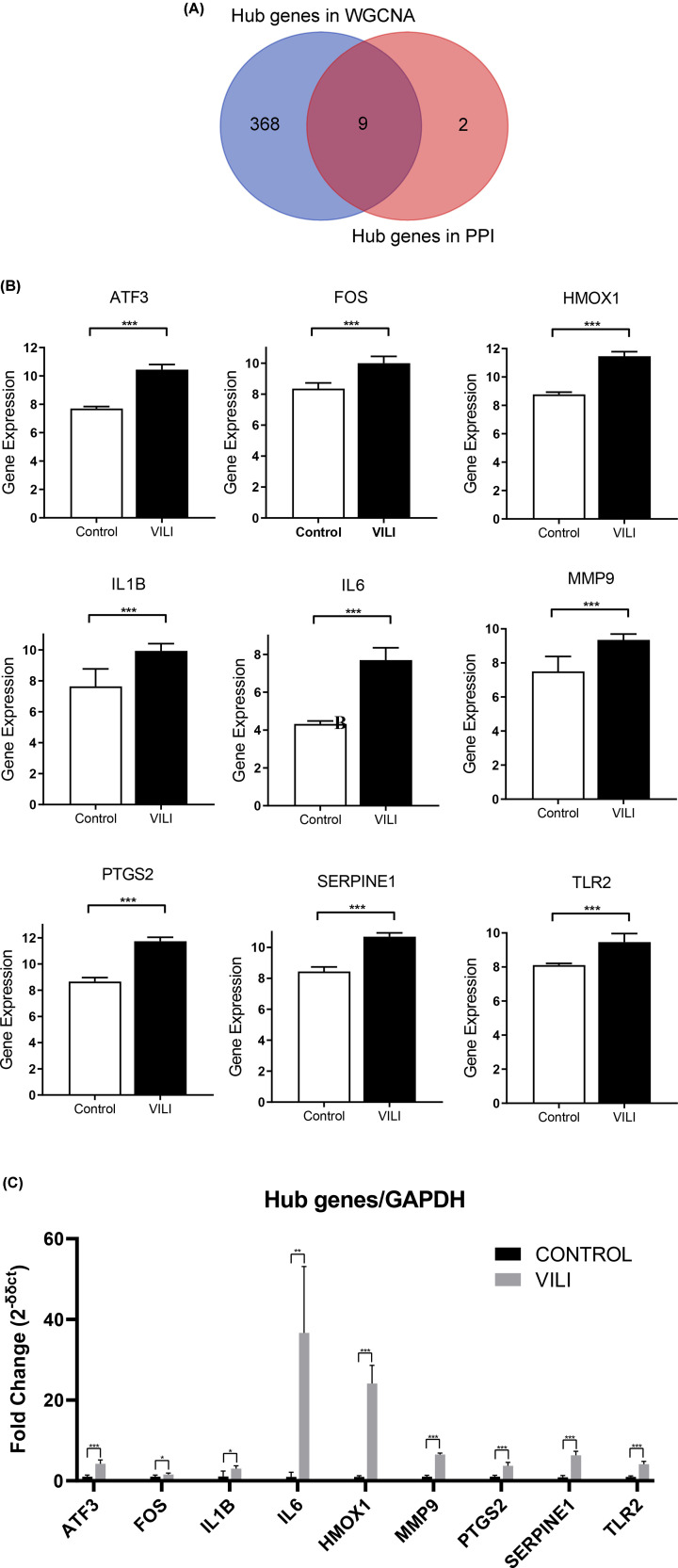
The identification and validation of final key genes of VILI (**A**) Overlap of WGCNA hub genes and DEGs hub genes. (**B**) The validation of identified key genes in GSE86229. (**C**) The validation of identified key genes by performing qPCR.

The hub genes in the darkolivegreen and blue modules and those screened out from DEGs were intersected and yielded nine key genes: Tlr2, Hmox1, Serpine1, Mmp9, Il6, Il1b, Ptgs2, Fos and Atf3 (Figure 7A). The expression of those nine genes were up-regulated in GSE86229 (Figure 7B). To confirm the expression levels of those nine key genes, qPCR was conducted using the total RNA extracted from lung samples from VILI and controls. The qPCR results showed that those nine key genes were all overexpressed in VILI lung tissues, which were consistent with the prediction results (Figure 7C). The aberrant expressions of these nine genes could contribute to the VILI pathogenesis and progression, and need to be further investigated.

## Discussion

Mechanical ventilation is widely adopted in patients with ALI or respiratory distress syndrome, as well as in surgeries involving general anesthesia [1]. However, a deeper exploration into the mechanical ventilation has revealed that although mechanical ventilation significantly improves air exchange and oxygenation in patients, it may also cause or even exacerbate lung injuries, thereby inducing VILI [16]. The pathophysiology of VILI involves the interaction of multiple mechanisms and remains largely unknown. Despite various genes identified as being involved in VILI, the gene network associated with the etiology of VILI has not been elucidated. In the present study, four datasets from the same platform, involving 15 controls and 15 VILI mouse models, were included. WGCNA and DEGs analyses were used to determine significantly up-regulated or down-regulated genes. Part of those genes were hub genes correlated with VILI, but their molecular roles in VILI pathogenesis remain far from clear. The identified genes with detectable expression could serve as potential therapeutic targets for VILI, and are worthy of further studies.

The molecular mechanism of inflammatory lung injury caused by mechanical ventilation is still a subject of debates, and any single molecular process has not yet sufficed to elucidate the complex VILI pathology. Due to the overlapping and idiosyncratic nature of the spectrum of VILI, and the sporadic nature of its genetic continuity, it is difficult to thoroughly investigate the molecular mechanism of VILI pathogenesis [17]. Traditional biological methods can only detect a single independent molecular signaling pathway; however, by virtue of the gene microarray technology, the transcriptome analysis can detect multiple molecular pathways involved in VILI. Recent studies have manifested that genome analysis based on microarrays would substantially contribute to improving our understanding of mechanisms of VILI [18,19]. Li et al. uncovered candidate genes associated with VILI by using Genome-Wide Association Studies (GWASs). GWAS usually only identifies statistically significant SNP loci, therefore leaving out those genes that are low in correlation effect and thus have higher *P*-values [20]. For complex diseases, susceptibility genes are often composed of various minor genes with low correlation effect, and such approaches are likely to fail to detect important information. WGCNA is based on entire gene modules rather than focusing on individual genes, and it uses soft thresholding instead of the standard hard thresholding to split modules. It, thus, provides a better reflection of the actual biological network and can be used to pinpoint the highly correlated gene modules; by identifying module eigengenes or hub genes, those co-expressed gene modules can be determined, and the modules associated with phenotypic traits can be identified. Such an approach is more consistent with biological processes, and could contribute in identifying therapeutic targets or candidate biomarkers. The clustering criteria of WGCNA have biological significance, and have been widely used in exploring molecular mechanisms of various diseases [21]; but WGCNA has not yet been adopted in studying the biomarkers, therapeutic targets and pathogenesis of VILI.

In the present study, a total of 5204 VILI-associated genes, whose variance was within first quartile, were screened from the combined datasets for WGCNA. Several pathogenic mechanisms associated with VILI were revealed through functional enrichment analysis of these modules, among which the blue and darkgreen modules were the most VILI-correlated and therefore, were considered to be the most significant modules. By the integration of hub genes from the two modules and DEG analysis, the following key genes were identified: TLR2, HMOX1, SEPRINE1, MMP9, IL6, IL1B, PTGS2, FOS, and ATF3.

As a member of the TLR family, TLR-2 is a transmembrane protein localized on the cell surface, and induces the release of inflammatory factors by mediating cell signaling [22]. It plays an important role in initiating the inflammatory and immune response, and maintaining innate immunity of the host [23]. The excessive inflammatory response involving multiple cytokines, inflammatory mediators and effector cells in the lung is an important cause that induces the occurrence and progression of VILI [24]. Therefore, the regulation of inflammatory signaling has become one of concerns in the prevention and treatment of VILI. Using two different mechanical ventilation strategies, Villar et al. found that TLR-2/4 expression was significantly up-regulated and pro-inflammatory cytokine synthesis was elevated in the lung tissue and plasma of mechanically ventilated mice with high tidal volume [25]. TLR-2 can form a homodimer with its receptor, and be activated by MD-2, a secretory factor, resulting in NF-κB pathway activation through recruiting IRAK by interacting with MYD88, and inducing the release of inflammatory factors [26]. Thus, it is reasonable that TLR2 may play a crucial role in the immune inflammatory response observed in VILI.

HMOX1 is one of the major members of the heme oxygenase (HO) family [27]. HO serves as a rate-limiting enzyme in the heme degradation pathway in which it catalyzes the oxidation of heme into bilirubin, carbon monoxide and free iron ions [28]. It comprises three subtypes: HMOX1 induced by oxidative stress, and constitutively expressed HMOX2 and HMOX3 [29]. HMOX1 is also known as heat-shock protein 32 (HSP32), and plays important roles in multiple stimuli and pathological conditions, such as heat, hypoxia, oxidative stress, apoptosis and mucosal damage [30]. Present studies on HMOX1 are mainly focused on the oxidative stress and hypoxic injury, such as the research by Synowiec et al., on the involvement of HMOX1 in the oxidative stress process in age-related macular degeneration [31], and the investigation by Cordova et al. on the relationship between HMOX1 and systemic lupus erythematosus in children [32]. The VILI pathogenesis largely involves biological hazard, but it has not yet been conclusively determined whether HMOX1 plays a role in its pathogenesis. The inspired oxygen concentration may affect the VILI development. Dunigan-Russell et al. exposed both the HMOX1 knockout (HO-1 KO) mice and adult wild-type controls to a hyperoxic condition, and evaluated the degree of lung injury by detecting the ratio between the right lung and body weight [33]. It revealed that the ratio increased significantly in HO-1 KO mice, compared with that in the controls, suggesting that the HMOX1 has some protective effect in hyperoxic conditions and could be involved in the VILI pathogenesis.

MMPs, known as stomatin, are zinc-dependent endopeptidases that degrade the components of extracellular matrix (ECM), including laminin, collagen and fibronectin [34]. So far 26 members of the MMP family have been discovered. MMP9 is one of the most important members, and can effectively degrade collagen IV in the endothelial basement membrane, thus impairing the endothelial barrier function [35]. As a well-known gene for participating in inflammatory response, MMP9 has been studied extensively on its correlation to VILI. Chen et al. reported that MMP9 degrades gelatin and type IV collagen in basement membranes, disrupts alveolar–capillary integrity, and leads to hyperpermeability of pulmonary edema [36]. The study by Doroszko et al., in which mechanically ventilated Wistar rats were randomized into the doxycycline-administered group and placebo-administered group, revealed that doxycycline could assuage VILI by suppressing MMP9 activity [37].

PTGS2, also known as COX2, is involved in the inflammatory process [38]. Back in 1991, Xie et al. [39] and Kujubu et al. [40], respectively, discovered in their studies an mRNA encoding protein and a cDNA encoding protein, which were proved later to be the same enzyme; it is highly expressed in inflammatory cells when activated, and is named as COX-2, an isozyme of already known COX-1. Huang et al. used the COX-2 inhibitor celecoxib to intervene in a mouse model of VILI, and detected the pulmonary vascular permeability and leakage, inflammatory leukocyte infiltration and pulmonary oxygenation to assess the severity of VILI [41]. It revealed that the celecoxib-intervened VILI mice model exhibited a significant decrease in the associated indices compared with the control [41]. In another study, Meng et al. constructed a VILI mice model by injecting endotoxin to induce ARDS, followed by mechanical ventilation [42]. The constructed model was intervened with parecoxib, a COX-2 inhibitor, to detect the ratio of arterial oxygen partial pressure to the fraction of inspired oxygen (PaO_2_/FiO_2_), dry-to-wet weight ratio of lung tissue, inflammatory factors in serum and bronchoalveolar lavage fluid (BALF), and histopathological indices of lung tissue. It showed that the PaO_2_/FiO_2_ ratio was increased significantly in the parecoxib-intervened model; the dry-to-wet weight ratio of lung tissue, the count of macrophages and neutrophils in BALF, the total protein and neutrophil elastase levels, the tumor necrosis factor (TNF)-α and interleukin-1β levels in BALF and serum, and the prostaglandin E2 (PGE2) level were reduced substantially; the activity of myeloperoxidase (MPO) activity and levels of malonaldehyde, Bax and COX-2 were decreased significantly; the survival rate of rats was improved. All these studies indicated that VILI could be effectively alleviated by inhibiting COX-2 activity.

ATF3 is an important member of the ATF/CERB family of transcription factors, and is extensively involved in the early stress response, apoptosis and tumorigenesis [43]. It also binds to the ATF/CRE site of macrophage inflammatory protein 1β (MIP-1β/CCL4) promoter to inhibit the expression and secretion of CCL4 in macrophages and control the excessive inflammatory response [44]. The role played by AFT3 in VILI has been investigated in various studies. By comparing the responses of ATF3-deficient and wild-type mice in an *in vivo* model of VILI; Akram et al. found that ATF3 expression and nuclear translocation increased in the lungs of wild-type mice after mechanical ventilation, whereas AFT3-deficient mice were more susceptible to mechanical ventilation with or without endotoxin inhalation, as evidenced by the increase in cellular infiltration and pro-inflammatory cytokines in the BALF, and by the increased extent of pulmonary edema and tissue damage [45]. Therefore, it can be inferred that ATF3 could balance and inhibit the high tidal volume-induced inflammation, and suppress its adverse effect on lung tissue. Shan et al. found that ATF3 protects against VILI by preventing inflammatory cell recruitment and barrier dysfunction in a cell-specific manner [46].

IL-6 and IL-1β are biologically active cytokines produced by activated monocyte/macrophages, and exert biological effects in inflammation and immunological diseases through multiple pathways [47]. Liu et al. reported that in VILI-induced inflammation, phosphorylated NF-κB could promote the synthesis of NLRP3 inflammasome, increase release of IL-1β, and aggravate lung inflammation by up-regulating NLRP3 expression [48].

Serpine1 is a component of the fibrinolytic system, and can directly act on endogenous plasminogen activator (PA) to reduce its activity and inhibit fibrin degradation [49]. PA is usually inhibited by specific inhibitors, the most common of which is PAI-1 [50]. Wolthuis et al. found that deficiency of PAI-1 gene reduces neutrophil’s entry into the alveoli during mechanical ventilation, suggesting that PAI-1 plays a stimulatory role in cell migration into the alveoli, and inhibition of PAI-1 may reduce the occurrence of VILI [51]. Considering the inhibitory effect of serpine1 on PA activity, we speculate that serpine1 could decrease the incidence of VILI and stem its progression.

FOS is a nuclear phosphoprotein encoded by mature mRNA transcribed from the c-fos gene [52]. It is an inherent gene in human or mammal cells, also known as immediate early response gene. As a transcription factor, FOS protein is an important regulator of cell growth, division, proliferation, differentiation and even programmed death [53]. FOS protein and c-fos have recently received lots of attention and been studied extensively. Fos protein can bind to c-Jun protein to form a dimer, which is an active transcription factor, AP-1 [54]. Imai et al. reported that AP-1 activation leads to IL-8 transcription, which recruits neutrophils in the lung, a critical step towards VILI development [55]. Therefore, we suggest that Fos protein can induce the production of large numbers of inflammatory factors by activating AP-1, thus promoting the VILI development [55].

There are still several limitations in the present study: firstly, the sample size included is far from substantial, and future studies are needed in which more ample samples are included to further confirm our results. Moreover, the samples included are all exclusively collected from lung tissues. Given that mechanical ventilation modeling allows inflammatory cells to flow into lung tissue, the exclusive use of samples derived from lung tissue in detection would have an impact on the validity of the test results, and thus on our understanding of the cellular origin of the target genes that regulate pathological changes. Data on samples from multiple sources including blood need to be included in future studies to improve our understanding of VILI-induced gene changes.

## Conclusion

In the present study, nine key genes with expression differences in VILI were screened by WGCNA by integrating multiple datasets. Those nine key genes were analyzed to study their potential functions and unique effects in VILI, in order to provides a reliable basis to relieve VILI.

## Data Availability

The data used and/or analyzed in the present study are available in the GEO database (https://www.ncbi.nlm.nih.gov/geo).

## Abbreviations

ALI: acute lung injury
ATF3: activating transcription factor
BALF: bronchoalveolar lavage fluid
DEG: differentially expressed gene
GS: gene significance
GWAS: genome-wide association study
HMOX1: heme oxygenase 1
MM: module membership
MMP: matrix metalloproteinase
PA: plasminogen activator
PaO_2_/FiO_2_: ratio of arterial oxygen partial pressure to the fraction of inspired oxygen
PPI: protein–protein interaction
PTGS2: prostaglandin-endoperoxide synthase 2
qPCR: quantitative PCR
TLR: toll-like receptor
TOM: topological overlap matrix
VILI: ventilator-induced lung injury
WGCNA: Weighted Gene Co-Expression Network Analysis
